# MMP-14 and CD44 in Epithelial-to-Mesenchymal Transition (EMT) in ovarian cancer

**DOI:** 10.1186/s13048-016-0262-7

**Published:** 2016-09-02

**Authors:** Maria Caroline Vos, Eva Hollemans, Nicole Ezendam, Harry Feijen, Dorry Boll, Brenda Pijlman, Hans van der Putten, Paul Klinkhamer, Toin H. van Kuppevelt, Anneke A. M. van der Wurff, Leon F. A. G. Massuger

**Affiliations:** 1 Department of Obstetrics and Gynaecology, Elisabeth-Tweesteden Hospital, PO Box 90151, 5000 LC Tilburg, The Netherlands; 2 Department of Pathology, Elisabeth-Tweesteden Hospital, PO Box 90151, 5000 LC Tilburg, The Netherlands; 3 Netherlands Comprehensive Cancer Organisation, Utrecht, The Netherlands; 4 CoRPS, Tilburg University, Tilburg, The Netherlands; 5 Department of Obstetrics and Gynecology, Amphia Hospital, Breda, The Netherlands; 6 Department of Obstetrics and Gynecology, Elisabeth Tweesteden Hospital, Tilburg, The Netherlands; 7 Present address: Department of Obstetrics and Gynaecology, Catharina Hospital, Eindhoven, The Netherlands; 8 Department of Obstetrics and Gynaecology, Jeroen Bosch Hospital, ‘s-Hertogenbosch, The Netherlands; 9 PAMM, Laboratory for Pathology, Eindhoven, The Netherlands; 10 Department of Biochemistry, Radboud Institute for Molecular Life Sciences, Radboud University Medical Centre, PO Box 9101, 6500 HB Nijmegen, The Netherlands; 11 Department of Obstetrics and Gynecology, Radboud University Medical Centre, PO Box 9101, 6500 HB Nijmegen, The Netherlands

**Keywords:** MMP-14, CD44, Epithelial-to-mesenchymal transition, Ovarian cancer

## Abstract

**Background:**

To investigate the expression of MMP-14 and CD44 as well as epithelial-to-mesenchymal transition(EMT)-like changes in ovarian cancer and to determine correlations with clinical outcome.

**Methods:**

In 97 patients with ovarian cancer, MMP-14 and CD44 expression as determined by immunohistochemistry was investigated in relation to EMT-like changes. To determine this, immunohistochemical staining of E-cadherin and vimentin was performed.

**Results:**

Patients with expression of both MMP-14 and CD44 in their tumors had a poor prognosis despite complete debulking. Serous histology in advanced-stage tumors (FIGO IIB-IV) correlated with CD44 (rho .286, *p* < 0.01). Also, CD44 correlated with percentage vimentin expression (rho .217, *p* < 0.05).

In logistic regression analysis with complete debulking as the outcome parameter, CD44 expression was found to be significant (OR 3,571 (95 % Confidence Interval 1,112–11,468) *p* = 0.032), though this was not the case for MMP-14 and EMT parameters.

**Conclusion:**

The subgroup of patients with double expression of MMP-14 and CD44 had a poor prognosis despite complete debulking. Serous subtype in advanced-stage patients and CD44 expression were found to be correlated with vimentin expression, and CD44 expression was found to be significantly correlated with complete debulking. However, a significant correlation between EMT and clinical parameters was not found.

## Background

In epithelial cancer, epithelial-to-mesenchymal transition (EMT) is an important pathophysiological process. EMT enables epithelial carcinoma cells to invade the underlying stroma. Usually, EMT occurs at the tumor’s invasive front and EMT can be recognized by its surrounding stromal reaction in conventional histopathology. Often, EMT is not complete and reversible and it is probably more correct to describe the process in the tumor as EMT-like changes [1].

In most other gynecological tumors, EMT-like changes are found [1]. In ovarian cancer however, the invasive front is less clearly defined than in other tumors of the female genital tract. Also, the relationship between EMT and prognosis has not been clearly established in ovarian cancer [1].

During EMT-like changes, the structure of the tumor cells is remodeled. Epithelial cells undergo a transformation from apical-basal to front-back polarity, and the actin cytoskeleton is restructured with a morphological change from a rounded epithelial feature to a spindle shape with mesenchymal characteristics. At the cell’s front and leading edges, lamellipodia are located, which are actin cytoskeleton protrusions of the cell responsible for its movement [2].

EMT-like changes result in down-regulation of epithelial markers such as E-cadherin and cytokeratins and increased expression of mesenchymal markers such as vimentin [1, 3, 4]. This is sometimes accompanied by an up-regulation of matrix metalloproteinases (MMPs) [1] and another important molecule in EMT, CD44 [5].

CD44 is a cell-surface molecule involved in intercellular interactions, cell adhesion and migration. In addition to the standard form of CD44, splice variants are described in the literature [6]. CD44 in its standard form located on the surface of ovarian cancer cells contributes to peritoneal metastasis by binding to the hyaluronan coat on mesothelial cells [7–9]. In the cell’s interior, CD44 is linked to the actin skeleton and thereby induces the transformations to spindle morphology in EMT-like changes [5].

MMPs also play a role in regulating EMT and thus in promoting invasion and metastasis. MMPs are ubiquitous zinc-dependent proteases that are important in numerous physiological and pathophysiological processes, including ovulation, reproduction, inflammation and especially arthritis and cancer. In cancer, various MMPs play an important role in progression and metastasis [10]. In general, the greater the overexpression of MMPs in primary and metastatic tumours, the worse the prognosis [11, 12]. In this study, we focused on MMP-14 [13, 14].

In EMT, MMP-14 and CD44 act jointly. The presence of both molecules at the lamellopodia’s edge has been demonstrated in cells undergoing EMT. [5] In vitro, MMP-14 and CD44 form a complex at the lamellipodia through the PEX domain in migrating cells [15].

When CD44 and MMP-14 are co-expressed in various cancer cell lines, they stimulate cell migration after the soluble part of CD44 is shed from the cell surface [16]. Various stimuli may trigger cleavage of the CD44 ectodomain, which is mediated by MMPs. This seems to indicate that CD44 links MMP-14 and the actin cytoskeleton in invasive cancer cells [5].

In vitro, cells overexpressing CD44 exhibit morphologic changes from epithelial to mesenchymal features with increased cell migration and invasion. CD44 overexpression in colon cancer cells has been shown to inhibit E-cadherin expression, whereas it induces the expression of EMT markers such as vimentin and MMP-14 [5].

The exact relationship between these molecules in the EMT-like process in ovarian cancer has yet to be determined. Also not yet known is how EMT and the expression patterns of EMT markers correlate with clinical outcome parameters such as debulking surgery and, ultimately, survival. In a regional cohort study with 5-year follow-up, the presence of MMP-14, CD44 and the EMT markers E-cadherin and vimentin was studied and correlated with the outcome of debulking surgery and survival.

## Methods

### Clinical data

From the regional Registration System Oncological Gynaecology (ROGY) all ovarian malignancies diagnosed between 1 January 2007 and 31 December 2008 were selected. This group consisted of 125 patients diagnosed with primary ovarian cancer at Elisabeth Tweesteden Hospital Tilburg, Amphia Hospital Breda, St. Catharine Hospital Eindhoven and Jeroen Bosch Hospital’s-Hertogenbosch, all in the Netherlands.

All patients were followed until 1 June 2013. Nine patients died before the diagnosis could be histopathologically confirmed. Seven patients were operated on in other hospitals and tissue of eight patients was sent to other hospitals for consultation or research; therefore, it was not available for this study. In the case of four patients, no tumor was found in the remaining paraffin-embedded tissue, leaving 97 patients for analysis.

All patients underwent a laparotomy with staging or debulking if such interventions were indicated by clinical stage and frozen-section results. Debulking surgery was found to be complete if no macroscopically visible or palpable tumor tissue was present. In patients with FIGO stage Ia or Ib ovarian cancer with differentiation grade I, no adjuvant therapy was given. All other patients received six courses of adjuvant platinum-based chemotherapy.

Data collection from the ROGY registry was prospective. Additional clinical and follow-up data were collected from the medical records. FIGO stage (I to IV) was categorized according to 2014 criteria [17]. Serum levels of CA-125 were determined pre-operatively.

Since MMP-expression is influenced by chemotherapy, only histopathological samples before chemotherapy were used.

### Histopathological data

All histopathological results were independently reviewed by two gynecopathologists (PK, AAW). Consensus on definitive results was reached in a joint consultation.

Histology and differentiation grade were categorized according to World Health Organization (WHO) criteria, the grade being assigned on the basis of the observer’s impression of both architectural and cytological features [18].

### Immunohistochemistry

From archives of the histopathology laboratories, paraffin-embedded tumour blocks were selected. Immunohistochemistry was performed as previously described [19]. In brief, sections (3 μ) were deparaffinized in xylene and rehydrated with graded alcohol. Each slide included positive controls for MMP’s consisting of placental tissue. Each run included negative controls without a primary antibody. Endogenous peroxidase was blocked with 3 % H_2_O_2_ and 5 % normal goat serum. After each incubation step, slides were washed twice with PBS. As primary antibody for MMP-14, a polyclonal antibody (Thermoscientific, Waltham, MA, USA) in a dilution of 1:20 with incubation at roomtemperature (RT) for 60 min was used. As a primary antibody for CD44, a polyclonal antibody (Proteintech, Manchester, United Kingdom) in a dilution of 1:80 with incubation at RT for 30 min was chosen. The primary antibody for E-cadherin was a polyclonal antibody (Proteintech, Manchester, United Kingdom) diluted 1:40 with incubation at RT for 30 min and for vimentin, a polyclonal antibody (Proteintech, Manchester, United Kingdom) diluted 1:80 at RT for 30 min was used. After washing with PBS, slides were incubated with a secondary antibody, poly-HRP-GAM/R/R IgG Powervision (Immunologic, Duiven, the Netherlands) for 60 min at room temperature. Staining was done with diaminobenzidine (Immunologic, Duiven, the Netherlands) in substrate buffer (20 μl/ml) for 5 min. Finally, the slides were counterstained with hematoxylin.

### Scoring of immunostaining

The scoring system used for MMP-14 incorporated staining intensity (0 = absent, 1 = weak, 2 = moderate, 3 = strong) and percentage of positive cells (0 = 0 %, 1 = 1–25 %, 2 = 26–50 %, 3 = 51–75 %, 4 = 76–100 % of cells). Stromal staining was recorded separately [13]. Points for intensity and percentage of positive tumor cells were added into an Overall Score (OS) according to Kamat [13], where 0 = no expression or < 5 % of cells positive, 1 = weak expression (1–2 points), 2 = moderate expression (3–4 points) and 3 = strong expression (5–6 points). The overall score was dichotomized into 0 for no/weak expression (1–2 points) and 1 for moderate-to-strong expression (3–6 points).

For immunostaining with CD44 and E-cadherin, the points for percentage of tumor cells stained and the intensity of staining were added and the tumors were dichotomized into two groups for each antibody: low expression (OS = 0) and high expression (OS = 1). Low expression included tumors with < 25 % staining, regardless of the intensity, and tumors with negative or weak intensity. High expression included tumors with at least 25 % staining and average-to-strong intensity.

Regarding EMT, a tumor was scored as positive (OS = 1) if vimentin was found to be expressed in at least 25 % of the epithelial tumor cells, regardless of the intensity and the expression pattern of other markers. For vimentin, the percentage of positive cells was also determined for each slide.

Two investigators scored all slides for immunohistochemistry (EH, MCV). If they disagreed, consensus was reached by consulting a gynaecopathologist (AAW).

### Statistical analysis

Statistical analysis was performed using the Statistical Package for Social Sciences 20.0 (SPSS Inc., Chicago, Il). Descriptive statistics were used to describe patient characteristics and immunohistochemistry scores of early-stage and advanced-stage patients. For normally distributed continuous variables, independent samples *t*-tests were used, whereas for not normally distributed continuous variables, Mann-Whitney tests were used. For categorical variables, chi-square tests were used.

To determine the correlation coefficients between MMP and EMT parameters, Spearman’s rho correlation coefficients were calculated; a value of .1 being considered small, .3 medium and .5 large.

A logistic regression was performed with complete or incomplete debulking as the outcome parameter and MMP-14 and EMT parameters as independent variables.

## Results

### Distribution of EMT-like changes within ovarian tumours

**Fig. 1:**
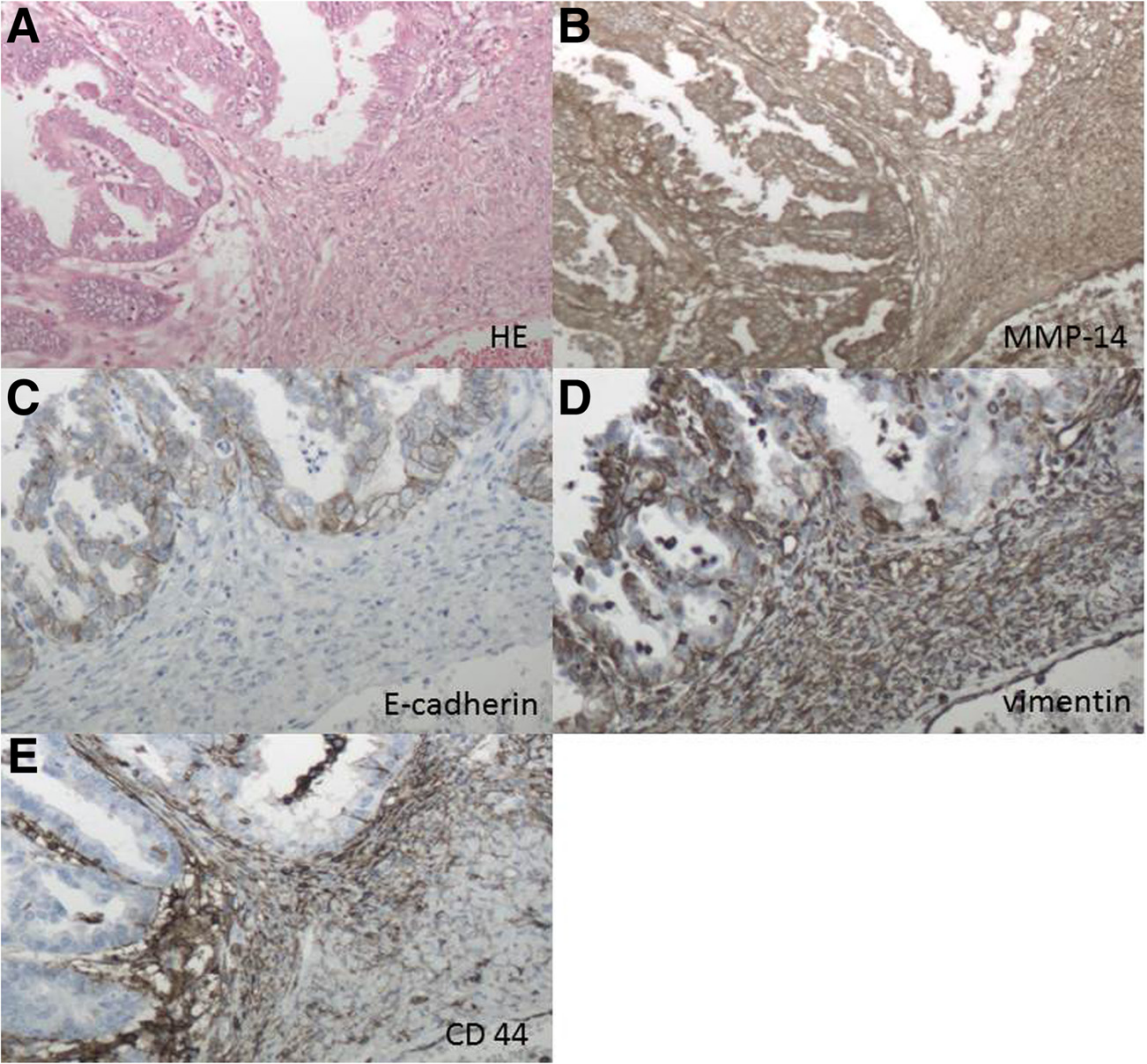
Immunohistochemistry panel of a clear cell carcinoma in a 56-year old patient in FIGO stage IA with survival of 62 months without recurrence. **a** haematoxylin-eosin, **b** MMP-14, **c** E-cadherin, **d** vimentin, **e** CD 44. Magnification 10 × 10

In Fig. 1, the immunohistochemical staining of all markers used in this study is shown in a clear cell carcinoma. This illustrates the typical expression patterns of the markers. MMP-14 is expressed both in the tumor epithelium and in the stroma, whereas E-cadherin is only expressed in the tumor epithelium. Vimentin is mainly found in the stroma and CD44 is also found in the tumor stroma, with increased expression close to the tumor epithelium.

**Fig. 2:**
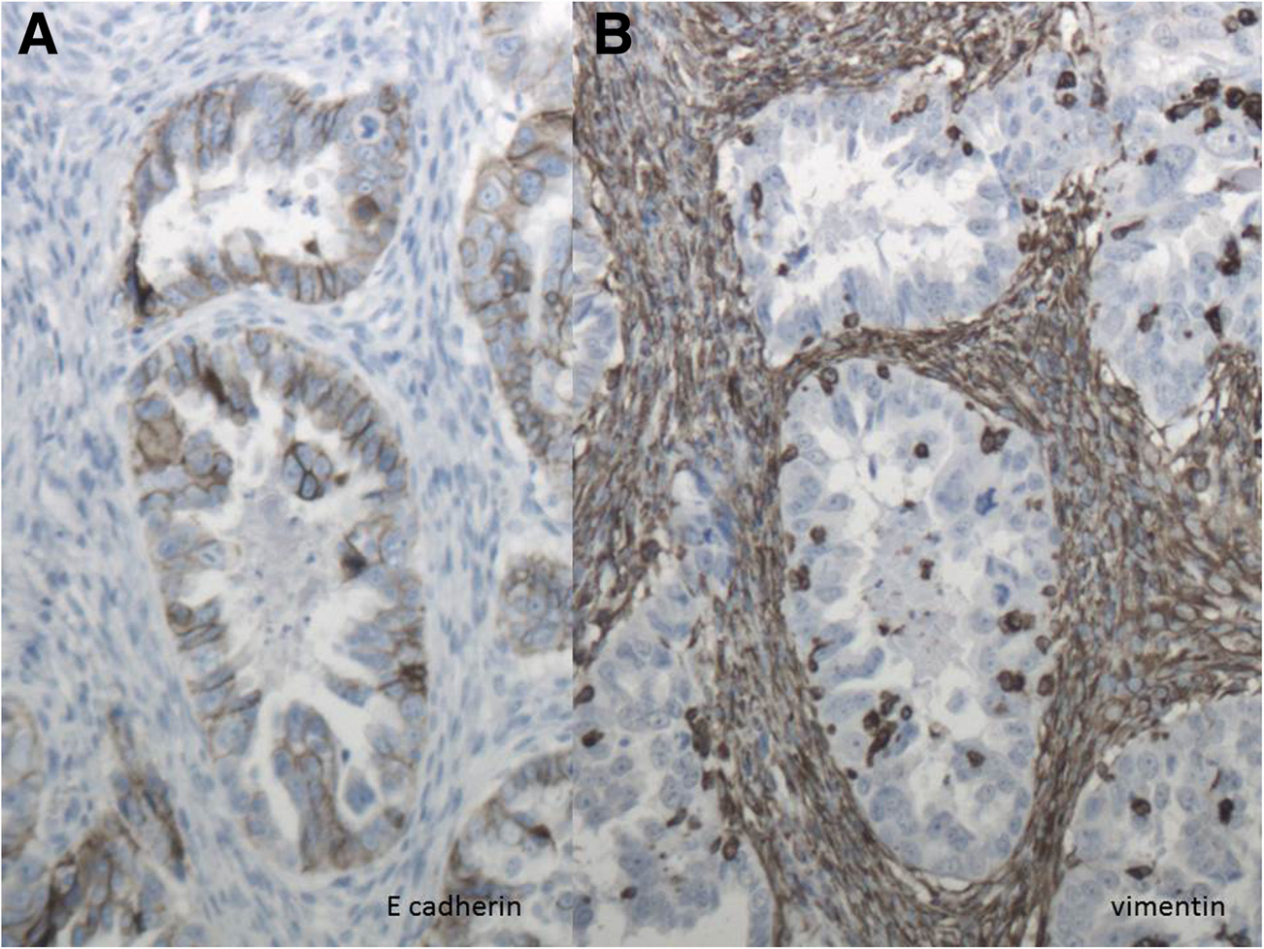
Immunohistochemistry of a high graded serous carcinoma in a 62-year old patient who underwent debulking surgery followed by chemotherapy. She recurred after 13 months and survived 41 months. The figure shows the distribution of positive staining for E-cadherin in the tumor epithelium and negative staining in the stroma (**a**). The second panel shows negative staining for vimentin in the epithelium and positive staining in the stroma (**b**). Magnification 10 × 10

Expression patterns for the various EMT markers are sometimes very heterogeneous. The first panel of Fig. 2 shows the distribution of positive staining of E-cadherin in the tumor epithelium and negative staining in the stroma (A). The second panel shows typical negative staining for vimentin in the epithelium and positive staining in the stroma (B) in an adjacent slide.

**Fig. 3:**
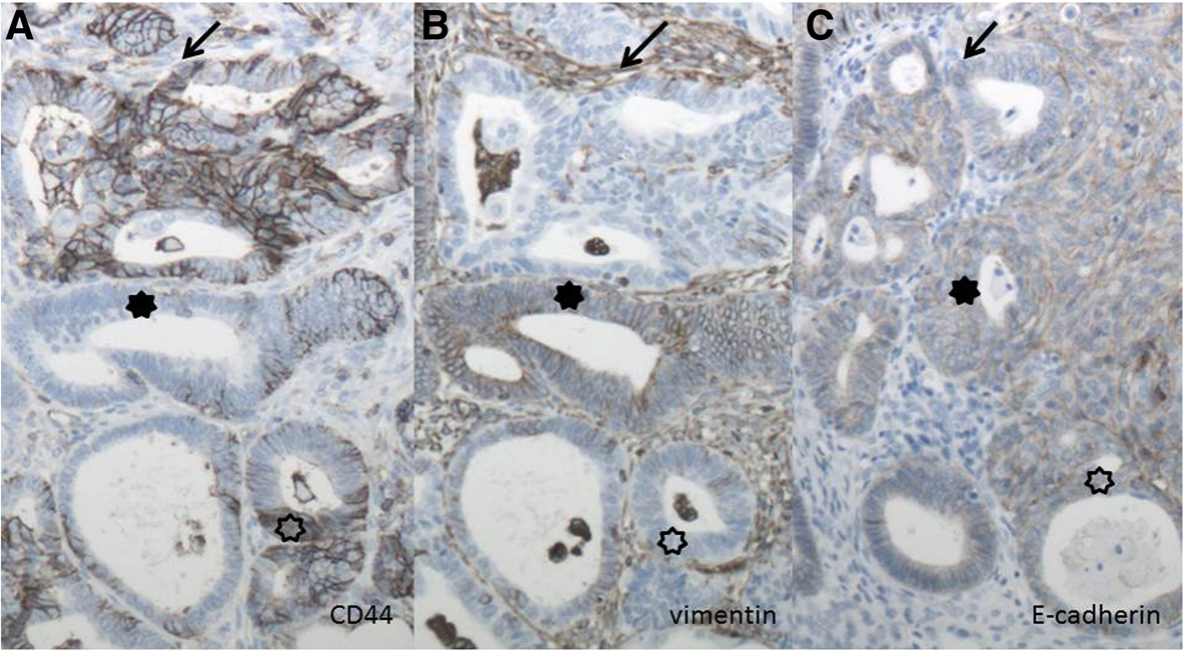
Immunohistochemistry of a well-differentiated endometrioid carcinoma in a 43-year old patient in FIGO stage IC who survived 73 months without recurrence. The tumor shows heterogeneity for CD44 (**a**), vimentin (**b**) and E-cadherin (**c**), negative epithelial staining for CD44 and vimentin and negative for E-cadherin and vice versa for stromal staining indicated with arrows. Negative staining for CD44 and vimentin and positive for E-cadherin indicated with closed asterisks. The adjacent stroma is either negative or only weakly stained. Open asterisks indicate positive epithelial staining for CD44 and E-cadherin with negative stromal staining and negative epithelial vimentin staining with positive stromal staining. Magnification 10 × 10

Figure 3 shows the heterogeneity of the various EMT markers within an endometroid tumor. In the tumor, epithelium close to the stroma shows positive staining for CD44 (A), whereas staining with vimentin (B) or E-cadherin (C) is negative at that site, this is indicated with arrows [1]. At other sites within the same tumor, the epithelium and stroma can exhibit positive or negative expression of both vimentin (B) and E-cadherin (C). Generally, E-cadherin expression is hardly evident in the stroma, whereas vimentin expression is abundant there. However, some epithelial areas express not only E-cadherin but also CD44 and vimentin.

### Immunohistochemistry

The patient characteristics are summarized in Table 1. The immunohistochemistry results are shown in Table 2. A significant difference was found in the expression of epithelial and stromal MMP-14 and CD44 between early-stage and advanced-stage patients, MMP-14 being more often expressed in the tumor epithelium in advanced-stage patients than in early-stage patients and less often in the tumor stroma. However, CD44 was less often found in the tumor epithelium in advanced-stage tumors, though one-third of the tumors expressed CD44 in the epithelium. No statistically significant difference between early and advanced-stage patients was found in the expression of E-cadherin and vimentin.

**Table 1:** Patient characteristics (numbers of patients unless stated otherwise)

	Early-stage (FIGO IA-Ic) (*n* = 23)	Advanced-stage FIGO (IIB-IV) (*n* = 74)	Total (*n* = 97)
Age (median (min-max))	59 (24–81)	64 (30–84)	62 (24–84)
Histological subtype			
Low-grade serous	10	14	24
High-grade serous	0	42	42
Mucinous	4	4	8
Endometroid	1	3	4
Clear cell	6	2	8
Adenocarcinoma unspecified		7	7
Mixed	2	2	4
Differentiation			
Low-grade	12	18	30
High-grade	11	56	67
CA 125 (median (min-max))	61 (7–3222)	615 (9–37500)	332 (7–37500)
FIGO stage			
IA-IC	23		23
IIB		14	14
III		44	44
IV		16	16
Debulking (only advanced stage disease)			
Complete		62	
Incomplete		12	
Overall Survival (months (minimum-maximum))	64 (28–75)	38 (0–76)	50 (0–76)
Number of deceased patients	4	51	55

**Table 2:** Results of immunohistochemistry and description of MMP-14/CD44 co-expression group

	Early-stage (*n* = 23)	Advanced-stage (*n* = 74)	*p* value*
MMP-14 Overall Score (number of patients)			0.07
0	8	11	
1	10	42	
MMP-14 stroma (number of patients)			0.03
no	4	25	
weak	14	23	
strong	0	5	
CD 44 Overall Score			0
0	6	47	
1	15	21	
E-cadherin Overall Score			n.s.
0	0	1	
1	21	67	
Vimentin epithelial score	30 %	32 %	n.s.
Group with co-expression MMP-14-CD44^a^	5	14	
Histological subtype			
Serous	1	9	
Mucinous	1	1	
Endometroid	1	0	
Clear cell	2	1	
Adenocarcinoma unspecified	0	2	
Mixed	0	1	
Grade			
Low-grade	1	3	
High-grade	4	11	
Debulking			
Complete		11	
Incomplete		3	
E-cadherin Overall Score			
0	0	1	
1	5	13	
Vimentin epithelial score	36 %	34 %	

*p value based on *T*-test and chi-square between early-stage and advanced-stage when appropriate

^a^numbers too small for statistical analysis, *n.s.* not significant

Additional chi-square tests were done to find out if these significant differences were attributable to histologic subtype and this was not the case (results not shown).

The subgroup of patients with both MMP-14 and CD44 expression differed from the group with no expression of both markers or expression of only one of these markers. Only 53 % of tumors with co-expression of MMP-14 and CD44 were of serous histology versus 72 % in the no/single-expression group; clear cell histology was 16 % versus 8 % in the co-expression group; and unspecified histology was 10 % versus 4 % in the adenocarcinoma group. Although complete debulking was high in the advanced-stage patients (11/14 (80 %)) with co-expression of MMP14 and CD44, the median overall survival was low (28 months versus 38 months in the whole group of advanced-stage patients). In this group, the expression of EMT markers E-cadherin and vimentin showed no differences with the expression in the whole group of patients (Table 2). Unfortunately, the group is too small for statistical analysis.

Table 3 shows the Spearman correlations between histologic subtype and MMP and EMT markers. The histological subtype correlated with CD44 expression (rho .286, *p* < 0.01). Subgroup analysis indicated that the significant correlation is determined by the correlation between serous histology in advanced-stage patients for CD44 (rho .284, p 0.007). Moreover, CD44 expression correlated with percentage vimentin expression (rho .217, *p* < 0.05).

**Table 3:** Correlations between histologic subtype and MMP-14 and EMT markers

	Histologic subtype	MMP-14	CD 44	E-cadherin
MMP-14	-0.13			
CDv44	0.286^b^	-0.07		
E-cadherin	-0.19	0.2	0.09	
Percentage vimentin	-0.05	-0.01	0.217^a^	-0.18

^a^Correlation is significant at the 0.05 level

^b^Correlation is significant at the 0.01 level

### Clinical outcome in terms of debulking surgery and survival

Logistic regression analysis with complete debulking as dependent variable and MMP-14 and EMT parameters as independent variables showed significance for epithelial CD44 expression (OR (Odds Ratio) 3,571 (95 % Confidence Interval 1,112–11,468) significance 0,032). MMP-14 expression was neither a confounder nor an interactor in this relationship.

For survival, no significance for MMP-14 expression nor for EMT parameters as E-cadherin or vimentin was found.

## Discussion

In this regional cohort with 5-year follow-up, the presence of MMP-14, CD44 and the EMT markers E-cadherin and vimentin and their interrelationships were investigated. The patients with double expression of MMP-14 and CD44 in their tumors had a poor prognosis despite complete debulking. Serous subtype in advanced-stage patients and CD44 expression were correlated with EMT markers and CD44 expression was found to be significantly correlated with complete debulking.

It is a challenge to interpret these findings pathophysiologically. A previous study found that CD44 expression is associated with peritoneal metastasis and EMT [16], but it is not clear how peritoneal metastasis and EMT are related to complete debulking. In our study, epithelial CD44 expression was a predictor for complete debulking, which is consistent with the better prognosis for CD44-positive tumors [20].

To determine CD44 expression in this study, a pan-CD44 antibody was used in order not to miss relevant CD44 expression in the tumors. Pan-CD44 expression is associated with a better prognosis in ovarian cancer, though some other isoforms of CD44 are associated with a poorer prognosis [7, 20, 21]. The first finding is consistent with the correlation of CD44 with complete debulking. These studies of somewhat older date correlated CD44-isoforms with prognosis in ovarian cancer: in a small series a low frequency of CD44v5 and CD44v6 were found [7]. In a larger series, CD44 was present in about half of the tumours and indicated a good prognosis [20]. In another series, CD44s, CD44v3 and CD446 were upregulated in ovarian cancer, but declined with advancing FIGO stage [21]. In a series with lymphnode metastasis, a high expression for CD44v5 and CD44v6 was found [22]. Recently, CD44v6 was found to correlate with prognosis while in another study, CD44v8-10 indicated a better prognosis and a more epithelial phenotype [23, 24]. A recent meta-analysis concluded that only CD44s is related to chemoresistance in ovarian cancer [25]. It may be that using an antibody for a specific isoform of CD44 would have resulted in a different relationship with EMT markers, because some isoforms of CD44 may be more important than others in EMT, like CD44v8-10.

However, this is not fully studied in ovarian cancer. In a study of colorectal cancer, the standard isoform CD44s seemed to be correlated with EMT, but higher CD44 expression predicted poor survival in that study in contrast to our results in ovarian cancer [26].

In other gynecological malignancies, the presence of EMT-like changes has been found to indicate poor prognosis [1]. Our study in ovarian cancer was not consistent with that finding.

A tentative explanation for this apparent discrepancy may be the pathology of ovarian tumors. Typically, a small proportion of ovarian tumors invade the ovary internally from their cystic lining, whereas the majority spread extensively on the peritoneum and to a lesser extent on the ovarian surface. Therefore, the invasive front is difficult to determine. Arguably, the superficial spreading on the peritoneal surface does not require the invasive properties of cells with EMT-like changes. However, deep invasion into other organs and metastasis into the liver for example do occur in ovarian cancer patients, albeit infrequently. In ovarian cancer, both changes to an EMT-like phenotype as well as a reverse to MET (Mesenchymal-to Epithelial Transition)-like changes are found [27].

The advantage of using an immunohistochemical panel for EMT, as in this study, is that various aspects of EMT in ovarian cancer are revealed.

One such aspect is the heterogeneous expression of the markers. The pattern is either positive expression of both E-cadherin and vimentin, negative expression of both these markers, or positive expression of one and negative expression of the other. Positive areas of E-cadherin expression indicate an epithelial phenotype, whereas positive expression of vimentin indicates a mesenchymal phenotype. Figure 2 shows an area where the expression patterns of E-cadherin and vimentin are heterogeneous. Although this heterogeneous pattern may be easy for the pathologist to interpret qualitatively, it makes it difficult to score the immunohistochemical results quantitatively, because in practice only the intensity and percentage of the cells are scored and heterogeneous expression is difficult to quantitate.

We studied primary ovarian tumor tissue. EMT markers in primary tumors may be expressed less than in metastatic tumors, especially in advanced-stage disease, as has been found for some CD44 isoforms. [22] It is the extent and progression of metastatic disease that eventually determines prognosis in a particular patient.

Although we have found that, for these reasons, EMT-like changes does not indicate prognosis in ovarian cancer, some of our results are consistent with the hypothesis that EMT is stimulated by CD44 and MMP-14 expression [5]. In the present study, the patients with double expression of MMP-14 and CD44 in their tumor had a poor prognosis. CD44 was found to be correlated not only with serous histology in advanced-stage patients but also with vimentin expression as a marker for EMT (Table 3). These correlations have a magnitude of 0.2-0.3, indicating a moderate effect. No correlation with EMT-like changes was found for MMP-14.

However, the small subset of tumors with both MMP-14 and CD44 expression seem to resemble type-I tumors with a lower percentage of serous histology and a higher percentage of clear cell and unspecified histology [28]. As previously stated, the choice of CD44 isoform to be measured may be important, as was described in a study on a small series of clear cell carcinomas where CD44-10v expression predicted recurrence and death [29].

The lack of significance of EMT for prognosis in ovarian cancer evident in the present study may also be due to the cross-sectional study design, in which the tumors were not followed in time. If a subset of the tumors with EMT-like changes were to result in a poorer prognosis for those patients, the statistical significance of this would be lost due to dilution with patients in which EMT is irrelevant to the clinical course. Solving this problem by collecting large series of tumor samples and performing immunohistochemistry on them requires preparation of such samples in one laboratory according to a uniform protocol. Bias may result in various ways, including missing samples and different protocols for tissue handling and fixation in different hospitals and laboratories. A shortcoming of our study was a relatively high number of missing samples for the various markers.

A strength of our study was the prospective data collection via the ROGY registry, which is used to register patients in all of the participating hospitals and sign them up for the tumor board meeting. By selecting patients via this registry and then collecting data from their hospital records, a thorough follow-up was achieved.

## Conclusion

From this cohort study on MMP-14 and CD44 expression in ovarian cancer, we conclude that the subgroup of patients with positive expression of both MMP-14 and CD44 had type-I tumors with poor prognosis despite complete debulking. We have demonstrated the presence and heterogeneity of EMT-like changes in ovarian cancer. We have also found that serous histology in advanced-stage patients and CD44 are correlated with vimentin expression and thus EMT-like changes. The most important finding of this study is that CD44 expression is positively related to complete debulking.

## Abbreviations

CD: Cluster of Differentiation
EMT: Epithelial-to-Mesenchymal-Transition
FIGO: International Federation of Gynaecology and Obstetrics
MET: Mesenchymal-to-Epithelial-Transition
MMP: Matrix MetalloProteinase
OR: Odds Ratio
OS: Overall Score
ROGY: Registration System Oncological Gynaecology
RT: Room temperature
SPSS: Statistical Package for the Social Sciences
